# Assessment of Host-Associated Genetic Differentiation among Phenotypically Divergent Populations of a Coral-Eating Gastropod across the Caribbean

**DOI:** 10.1371/journal.pone.0047630

**Published:** 2012-11-02

**Authors:** Lyza Johnston, Margaret W. Miller, Iliana B. Baums

**Affiliations:** 1 University of Miami, Rosenstiel School of Marine and Atmospheric Science, Miami, Florida, United States of America; 2 National Oceanic and Atmospheric Administration, National Marine Fisheries Service, Southeast Fisheries Science Center, Miami, Florida, United States of America; 3 The Pennsylvania State University, Department of Biology, University Park, Pennsylvania, United States of America; Natural History Museum of Denmark, University of Copenhagen, Denmark

## Abstract

Host-associated adaptation is emerging as a potential driver of population differentiation and speciation for marine organisms with major implications for ecosystem structure and function. *Coralliophila abbreviata* are corallivorous gastropods that live and feed on most of the reef-building corals in the tropical western Atlantic and Caribbean. Populations of *C. abbreviata* associated with the threatened acroporid corals, *Acropora palmata* and *A. cervicornis*, display different behavioral, morphological, demographic, and life-history characteristics than those that inhabit other coral host taxa, indicating that host-specific selective forces may be acting on *C. abbreviata*. Here, we used newly developed polymorphic microsatellite loci and mitochondrial cytochrome b sequence data to assess the population genetic structure, connectivity, and demographic history of *C. abbreviata* populations from three coral host taxa (*A. palmata*, *Montastraea* spp., *Mycetophyllia* spp.) and six geographic locations across the Caribbean. Analysis of molecular variance provided some evidence of weak and possibly geographically variable host-associated differentiation but no evidence of differentiation among sampling locations or major oceanographic regions, suggesting high gene flow across the Caribbean. Phylogenetic network and Bayesian clustering analyses supported a hypothesis of a single panmictic population as individuals failed to cluster by host or sampling location. Demographic analyses consistently supported a scenario of population expansion during the Pleistocene, a time of major carbonate reef development in the region. Although further study is needed to fully elucidate the interactive effects of host-associated selection and high gene flow in this system, our results have implications for local and regional community interactions and impact of predation on declining coral populations.

## Introduction

Although coral reefs are among the most biologically diverse ecosystems on the planet, the magnitude of this diversity and the mechanisms that drive and maintain it are still poorly understood [1]–[3]. In similarly diverse terrestrial ecosystems, it is estimated that 20%–40% of all animal species are specialist phytophagous insects [4]. Ecological niche partitioning has emerged as a prevalent mode of diversification for these insect herbivores and parasites [5],[6]. This process appears to be a dynamic continuum beginning when a subpopulation occupies a new host or habitat, often in response to some ecological trade-off such as reduced intraspecific competition or enemy free space [7]. Subsequent adaptation to the new host may then lead to reduced gene flow through selection for adaptive traits [8]–[10]. For speciation to occur, host-associated selection must be strong enough to overcome the potentially homogenizing effect of dispersal and gene flow from the original population [11], [12].

Similar to phytophagous insects, corallivores are ubiquitous members of coral reef communities that provide a link from foundational scleractinian coral species and their symbionts to higher trophic levels [13]. They range from generalist facultative consumers to host-specific obligate coral parasites [14], [15]. However, whereas the role of plant-herbivore interactions in the evolution and ecology of terrestrial ecosystems is the subject of a vast literature, relatively little is known about the interactions among corals and their natural enemies. If similar mechanisms of resource-associated ecological speciation are occurring on coral reefs, these coral-associated groups may harbor a large amount of cryptic biodiversity that has yet to be discovered [16],[17]. For instance, Gittenberger and Gittenberger [18] recently reported a large, cryptic, adaptive radiation of 14 Coralliophilid species in the genus *Leptoconchus* that are associated with mushroom corals (Scleractinia, Fungiidae) in the Indo-West Pacific. Many of these species are found in the same geographical area and can only be distinguished based on host association and molecular data. On the other hand, Oliverio & Mariottini [19] found no genetic differentiation between populations of *Coralliophila meyendorfii* that displayed host-specific size structure. Further studies are needed to elucidate the life-history characteristics and environmental conditions that facilitate or oppose host-specific differentiation and speciation for these and other coral associated organisms.

*Coralliophila abbreviata* are found on reefs throughout the Caribbean and tropical western Atlantic. These snails live and feed on the tissue of at least 16 species of scleractinian coral from five different families representing diverse growth forms and life-histories [20]. Different coral host taxa, therefore, likely provide variable food and habitat resources and selective regimes for *C. abbreviata*. Supporting this assertion, populations of *C. abbreviata* display host-specific behavioral, morphological, demographic, and life-history characteristics across the Caribbean. Snail populations found on the branching acroporid corals, *Acropora palmata* and *A. cervicornis*, are larger [21]–[24], due to increased growth [24], [25] and longevity [24], than on several massive and plating corals. Feeding mode and tissue consumption rate also vary among coral host taxa; snails on massive and plating corals generally behave more like ectoparasites, remaining relatively sedentary along the tissue margin where they do not create discernible feeding scars [21],[25]. Snails feeding on the acroporid corals, however, move up from the base of the coral colony, rapidly consuming tissue and creating conspicuous white feeding scars [21],[25]. Furthermore, these protandrous hermaphrodites change sex later at much larger sizes when residing on acroporid corals than on two other host coral species investigated [24]. These reports suggest that host-specific selective forces are acting on *C. abbreviata*. However, high dispersal and gene flow via planktotrophic veliger larvae with a putative pelagic larval duration (PLD) of more than 30 days (Johnston, unpublished data), may preclude host-specific adaptation and differentiation. We can thus use this system to investigate the interactive effects of potentially diversifying selection and homogenizing gene flow in the marine environment.

Here, we assess the Caribbean wide population genetic structure of *C. abbreviata* using de novo microsatellite markers as well as mitochondrial DNA sequence data. Our overall objective was to characterize the neutral genetic variation of *C. abbreviata* populations from different coral host taxa and geographical locations to assess a.) potential host-associated genetic differentiation, b.) the scale and patterns of gene flow across the Caribbean, and c.) the possible role of historical demographic fluctuations in shaping the observed patterns of genetic variation and population structure. An understanding of these processes is necessary to elucidate contemporary community interactions and to predict the potential impact of *C. abbreviata* on the persistence and stability of threatened host corals in the future. Further, this study contributes to the general understanding of the ecological and evolutionary processes that create and maintain biodiversity on coral reefs.

## Materials and Methods

### Sample Collection and Processing

**Figure 1:**
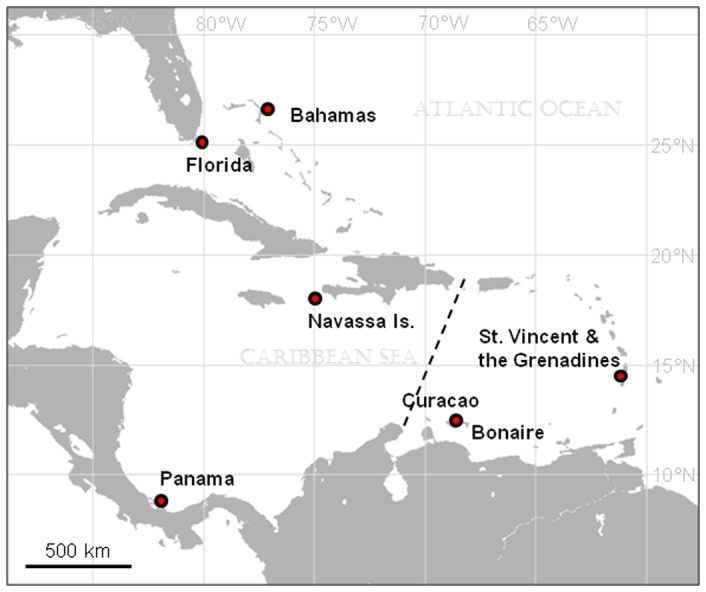
Map of sampling localities across the greater Caribbean. Dashed line represents the major regional break tested, between the eastern and western Caribbean.

Individual *Coralliophila abbreviata* were collected using SCUBA over a five year period (2001–2006) from three coral host taxa, 18 reef sites (reef names and coordinates can be found in Table S1) and six localities spanning most of the species' range (Fig. 1; Table 1). The primary coral host taxa sampled were *Acropora palmata* (ACR) and *Montastraea* spp. (MON). In Panama, however, *C. abbreviata* were not encountered on surveyed *A. palmata* and *Montastraea* spp. coral colonies and were thus collected from *Mycetophylia* spp. (MYC) corals, on which they were prevalent. All necessary permits were obtained for the described field studies, including: recreational saltwater fishing license issued to LJ by the Florida Fish and Wildlife Conservation Commission (Florida: Little Grecian and Sand Island); Special use permit 41529-2006-03 (Navassa: NW Point, W Pinnacles, DOT 118); ANAM permit PETCA 58323 (Panama: Hospital Pt.); general collection permit issued to CARMABI Foundation by the Dutch Antillean Government (Curacao and Bonaire: Taylors Made, Awa Blanca, Blue Bay, Playa Largu, Sea Aquarium); permit from the Department of Fisheries, granted to IBB (St. Vincent and the Grenadines: Blue Lagoon, Bequia, Conouan, Mustique, Tobago Cay, Union Island); permission granted to IBB from the Bahamian Department of Fisheries (Bahamas: Green Turtle Cay). No other permits were required for collections.

**Table 1:** Sample sizes of *Coralliophila abbreviata* by coral host and locality for mitochondrial cytochrome b sequences (mtDNA) and microsatellite markers (Msats).

Region	Locality	Coral host	Code	mtDNA	Msats
**West**	Florida	*A. palmata*	FL ACR	12	37
		*Montastraea* spp.	FL MON	15	34
	Bahamas	*A. palmata*	BAH ACR	–	12
	Navassa	*A. palmata*	NAV ACR	16	16
		*Montastraea* spp.	NAV MON	12	17
	Panama	*Mycetophylia* spp.	PAN MYC	30	51
**East**	St. Vincent & the Grenadines	*A. palmata*	SVG ACR	13	36
		*Montastraea* spp.	SVG MON	14	44
	Curacao/Bonaire	*A. palmata*	CUR ACR	–	25
		*Montastraea* spp.	CUR MON	18	39
**Total**				130	311

After collection, shells were crushed with a hammer and snail tissues were placed in 70%–95% ethanol and stored at −80°C until processing. Genomic DNA was then extracted from the foot tissue of individual *C. abbreviata* using either a standard CTAB extraction protocol or a Qiagen DNeasy Tissue Kit, following the manufacturer's instructions. Published primers (UCYTB151F and UCYTB270R) and PCR conditions were used to amplify a portion of the mitochondrial cytochrome b gene (*cyt b*; [26]). PCR products were purified using a Montage PCR Cleanup Kit (Millipore) and shipped to Elim Biopharmaceuticals (Hayward, CA, U.S.A.) for sequencing. Forward and reverse sequences were then assembled and edited in SeqMan and aligned using MegAlign (both DNASTAR, Inc).

Genomic DNA from two individual *C. abbreviata* was used to create a cDNA library enriched for microsatellite loci using a hybridization/capture technique modified from Glenn and Schable [27]. Thirteen primer pairs were designed, and after initial testing, five polymorphic microsatellite markers were found suitable for population genetic analyses (see Supporting Information S1).

### Characterization of Genetic Variation

Genetic diversity estimates for *cyt b* sequences, including the number of haplotypes, haplotype diversity (*h*) and nucleotide diversity (π) were calculated for all sampled populations using the program ARLEQUIN v. 3.5 [28]. The genealogical relationships among *cyt b* haplotypes were assessed by constructing a phylogenetic network using the median joining algorithm implemented in NETWORK v. 4.6 [29] with default values.

For each microsatellite locus, the number of observed alleles, allele frequencies, and observed and expected heterozygosity were determined using the program GENEPOP v.4 [30]. GENEPOP was also used to calculate *F*_IS_ values and test for deviations from Hardy-Weinberg equilibrium (HWE) and linkage equilibrium for each locus. All microsatellite loci were checked for the presence of null alleles and errors due to stuttering and large allele dropout using the program MICROCHECKER [31].

### Population Genetic Structure

Population differentiation was assessed with analysis of molecular variance (AMOVA), pairwise *F*-statistics, and exact tests of population differentiation using both mtDNA sequences and microsatellite data in ARLEQUIN v. 3.5. Due to small sample sizes from several reef sites (Table S1), individuals were grouped by localities, which thus represent the smallest geographical scale of comparison here (Table 1; Figure 1). For microsatellite data, locus-by-locus AMOVA was performed. The significance of all AMOVA tests was assessed with 16,000 nonparametric permutations. Exact tests of population differentiation included 10,000 dememorisation steps followed by an additional 100,000 Markov chain steps.

To test the hypothesis of host-associated differentiation, individuals from all localities were first pooled by coral host (ACR and MON) for analysis. Then, to tease apart potential effects of host and locality on measures of differentiation, populations were defined by host and locality (FL ACR, FL MON, BAH ACR [msats only], NAV ACR, NAV MON, SVG ACR, SVG MON, CUR ACR [msats only], and CUR MON; see Table 1 for code definitions) and grouped by either host or locality in hierarchical AMOVAs. The single sample collected from *Mycetophylia* spp. host corals in Panama (PAN MYC) was excluded from initial AMOVA tests of host-associated differentiation to avoid introducing error due to limited sampling. However, for exploratory purposes, we repeated the above tests including the PAN MYC sample.

Previously, Baums et al. [32] found regionally isolated populations of the host coral, *Acropora palmata* from the eastern Caribbean, delineated by the Mona Passage (between Hispaniola and Puerto Rico) and including the Lesser Antilles, and the western Caribbean including the Florida peninsula (Figure 1). This genetic break has been found in other coral reef organisms [33] and is consistently recovered in biophysical models of the region [34]–[36]. We thus tested for this break in *C. abbreviata* by pooling all individuals by these oceanographic regions (East and West; Table 1). Individuals were then pooled by locality (FL, BAH [msats only], NAV, SVG, CUR, PAN) and a hierarchical AMOVA was conducted to assess population genetic structure between regions and among localities within regions.

Further, to test for isolation by distance (IBD), Mantel tests were performed for both mtDNA and microsatellite data sets in ARLEQUIN. The geographical distance matrix was constructed using the shortest distances between locations via major ocean surface currents (as reported by [37]), measured using Google Earth. The significance of correlations was tested with 10,000 permutations.

To assess the power of the microsatellite data set to detect low levels of population differentiation, simulations were conducted in POWSIM v.4.1 [38] using the sample sizes for the various levels of structure tested in this study. The effective population size when drifting apart (*N*_e_) was set to 3,000 while the number of generations of drift (*t*) was varied to determine the lowest level of differentiation [*F*_ST_] that could be detected in simulated populations with at least 90% accuracy. With a *N*_e_ greater than 2,000, there should be little to no effect of loss of alleles on the power estimates for small *F*_ST_ values [38]. For each simulation, 100 replicates were run and significance was determined using Fisher's exact test.

Finally, the Bayesian Markov Chain Monte Carlo (MCMC) clustering method implemented in the program STRUCTURE v. 2.3 [39] was used to infer population structure using the microsatellite data set. This program approximates, *ad hoc*, the number of discrete populations (*K*) represented in a sample. It assigns individuals to populations and can identify migrants when prior population information is used. Here, the admixture, location prior (LOCPRIOR; [40]), and correlated allele frequencies [41] models were implemented. These models were chosen because, due to the high dispersal potential of the planktotrophic veliger larvae of *C. abbreviata*, populations are likely to have a common or an admixed ancestry and high gene flow. The selected models improve the clustering performance of STRUCTURE over other models in such situations where the signal of actual genetic structure may be weak, but do not tend to infer structure where there is none [40],[41]. Simulations were run for values of *K* from 1–10, with ten replicates per *K* value. All simulations were run with a burn-in length of 10^5^ steps followed by 10^6^ steps of data collection. The log posterior probability of the data (lnP[K]) was averaged across replicates for each *K* value to estimate the most likely number of populations using Structure Harvester v 0.6.6 [42].

### Demographic History Analyses

Historical demographic trends were investigated with several distinct methods. First, the mismatch distribution, based on the number of observed nucleotide differences between pairs of mitochondrial *cyt b* sequences was compared to the distributions expected under models of pure demographic expansion [43] and sudden spatial expansion [44] in ARLEQUIN. Model parameters (θ_0_, and θ_1_, and τ) were estimated by a generalized non-linear least-square approach with confidence intervals obtained through parametric bootstrapping (10^5^ replicates; [45]). For haploid, maternally inherited mitochondrial DNA, θ = 2*N*_e_μ, where *N*_e_ is the female effective population size and μ is the mutation rate. The time scale parameter (τ) is in mutational units; τ = 2*ut*, where *t* measures time in generations and *u* is the sequence mutation rate. The sums of squared deviations (SSD) of bootstrapped replicates were used to calculate the significance of the fit between the observed and expected mismatch distributions [45]. To convert the time since expansion (τ) from mutational units to years, we used mutation rates of 0.6% and 1.0% per site per MY based on fossil calibrated estimates of mtDNA sequence divergence rates between geminate species of mollusks in the Caribbean and Eastern Pacific [46], and a female generation time of 6 years, based on estimates of the age at which individuals change sex from male to female [24].

Second, Tajima's *D* [47] and Fu's *F*_S_ [48] statistics were calculated for *cyt b* sequences to test for deviations from selective neutrality and to refine inferences of demographic history, using ARLEQUIN. These statistics are expected to be zero for populations of constant size and in mutation drift equilibrium. Significant deviations from neutrality may be caused by selection or historic demographic fluctuations such as population bottleneck and expansion [48],[49]. Fu's *F*_S_ has been shown to be a particularly sensitive statistic for detecting sudden demographic expansion [48],[50]. Statistical significance was assessed by comparing the observed statistic values to expected values based on 10^5^ neutral coalescent simulations.

Next, we used the coalescent-based approach implemented in the program BEAST v1.6.1 to construct a Bayesian skyline plot (BSP; [51],[52]). Bayesian skyline analysis provides an estimate of the population size through time by sampling the posterior distributions of model parameters. The HKY + G model of nucleotide substitution (determined using the Akaike information criterion implemented in jModelTest v.0.1.1; [53]) was used with four gamma categories, estimated base frequencies, two codon partitions ([1+2], 3), and unlinked substitution rate parameters. A strict molecular clock was enforced with a rate of 1×10^−8^ substitutions per site per year. We chose the faster rate estimate as the conservative estimate for questions regarding the influence of historical events on contemporary genetic structure and gene flow. Operators were auto optimized. We ran three independent runs of 200 million MCMC steps sampled every 1000 steps after a 10% burn-in. The log and tree files for the three independent runs were combined using LOGCOMBINER v.1.6.1, discarding the burn-in and re-sampling every 1000 steps. Convergence and effective sample sizes (ESS) were evaluated in TRACER v1.5. After confirming that parameters showed good convergence and all ESS values were greater than 200, the BSP was constructed using the combined files in TRACER v1.5.

## Results

### Genetic Variation

**Figure 2:**
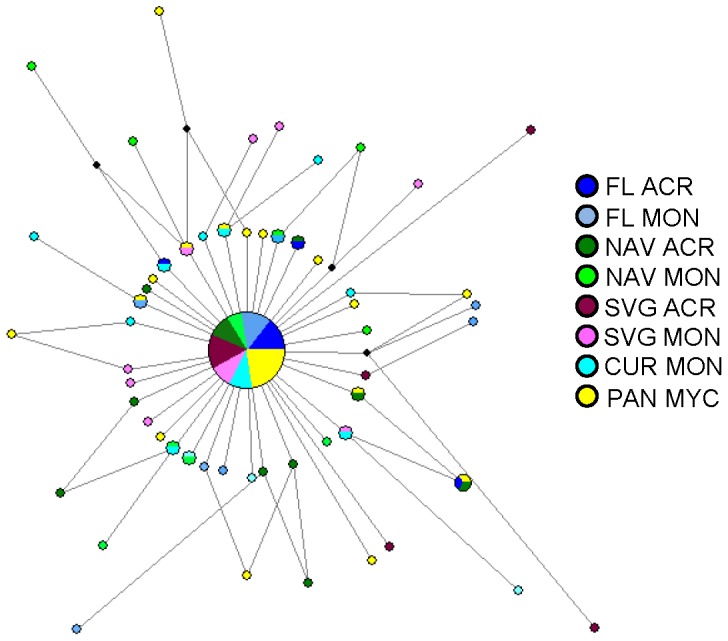
Median joining network for *cyt b* haplotypes from a sample of 130 *Coralliophila abbreviata*. Circles represent individual haplotypes. The size of the circle is proportional to the frequency of the haplotype in the sample and branch lengths are proportional to the number of mutational steps (range: 1–3). Small black circles represent missing/theoretical haplotypes.

A 366 bp fragment of the mitochondrial *cyt b* gene was sequenced and analyzed for 130 *Coralliophila abbreviata* individuals. The sequence alignment contained 55 polymorphic sites, resulting in 57 unique haplotypes. Haplotype diversity (*h*) was moderate to high across localities, ranging from 0.613 to 0.902 (global *h* = 0.773) and nucleotide diversity (π) was low across populations, ranging from 0.003 to 0.005 (global π = 0.004; Table 2). One ancestral haplotype, representing 48% (*n* = 62) of all observed haplotypes, was shared among all populations and was the most common haplotype found in each population. Eighty-four percent of the divergent haplotypes were singletons and separated from the ancestral haplotype by only 1–3 mutational steps, resulting in a star-like haplotype network (Figure 2).

**Table 2:** Genetic diversity indices, neutrality test statistics, and mismatch distribution parameters for mitochondrial *cyt b* sequences of *Coralliophila abbreviata* collected from Florida (FL), Navassa (NAV), St. Vincent and the Grenadines (SVG), Curacao (CUR), Panama (PAN), as well as all individuals combined (GLOBAL).

		Sampling locality					
		FL	NAV	SVG	CUR	PAN	GLOBAL
Genetic diversity	*N*	27	28	27	18	30	130
	*N_h_*	11	19	13	13	17	57
	*N_p_*	15	18	19	14	20	55
	*h*	0.612	0.881	0.701	0.902	0.791	0.773
	π	0.003	0.005	0.004	0.005	0.004	0.004
Neutrality tests	*D*	−2.46**	−2.13*	−2.43**	−2.14*	−2.44*	−2.64**
	*F_S_*	−8.37**	−20.70**	−9.50**	−11.80**	−17.12**	−28.01**
Mismatch dist.	τ	1.64	1.91	1.85	1.82	1.58	1.62
	θ_0_	0.040	0.004	0.417	0.000	0.000	0.018
	θ_1_	2.066	9999	2.848	9999	9999	15.025
	*P* (SSD)	0.970	0.503	0.975	0.330	0.960	0.997
	Rg	0.034	0.068	0.018	0.109	0.043	0.034
	*P* (Rg)	0.979	0.315	0.995	0.187	0.677	0.795

Genetic diversity indices: *N*, sample size; *N*_h_, number of haplotypes; *N*_p_, number of polymorphic sites; *h*, haplotype diversity; *π*, nucleotide diversity. Neutrality statistics: *D*, Tajima's statistic (Tajima 1989); *F*_S_, Fu's statistic (FU 1997). Mismatch distribution: *τ* (tau), time since beginning of expansion in mutational units; θ_0_ and θ_1_, initial and final population size estimators, respectively; *P* (SSD), probability of sum of squared deviations; Rg, raggedness statistic (Harpending 1994); *P* (Rg), probability of Rg.

* *P*<0.01.

** *P*<0.001.

Characteristics of each of the five polymorphic microsatellite loci, including the number of observed alleles, observed and expected heterozygosity, *F*_IS_ value, and the probability of deviation from HWE, are reported in Table 3. No significant (*α = *0.05) linkage disequilibrium between pairs of loci was detected and no loci deviated significantly from HWE after correction for multiple comparisons.

**Table 3:** Global characteristics of five polymorphic microsatellite loci for *Coralliophila abbreviata*.

Locus	Primer sequence (5′-3′)	Repeat motif	Size range (bp)	*N*	*N*_a_	*H*_O_	*H*_E_	*F*_IS_	*P*_HW_
Ca600	F: AAGGCAGAGGGGAAAACAGT	(CAT)_17_	181–235	300	20	0.867	0.868	0.005	0.45
	R: TTACCTGGGGACAACTGGAG								
Ca601	F: GAGCAGGGTGAAGAAAGACG	(AAG)_23_	210–401	289	67	0.927	0.978	0.046	0.04
	R: ACCCCTGCAAATTCTCCTTT								
Ca608	F: CTCCTTTCGTCTGGCTATGTG	(GT)_26_	179–253	299	35	0.926	0.936	0.016	0.27
	R: TAATGGGCAGTGGCAATTTT								
Ca609	F: TTGGTGTTTGTAGGTTTTTGTTC	(CT)_22_	178–264	293	50	0.952	0.974	0.021	0.03
	R: AAAAAGGGAGGGAAAGCAAA								
Ca612	F: TGGGACAGATGCACAGGTAA	(GT)_33_	291–382	298	48	0.940	0.960	0.022	0.03
	R: TTCAGCAGCGAAAGGTATCA								

Shown, for each locus, are the forward (F) and reverse (R) primer sequences, repeat motif, size range of alleles in base pairs (bp), global sample size (*N*), number of observed alleles (*N*_a_), observed (*H*_O_) and expected (*H*_E_) heterozygosities, fixation index (*F*_IS_), and uncorrected *P*-value for test of departure from Hardy Weinberg Equilibrium (*P*_HW_). Loci correspond to GenBank accession numbers HM156485, HM156486, HM156490–HM156492.

### Population Genetic Structure

While over 98% of the genetic variation was attributed to the within-population source of variation for all AMOVA analyses, there was some evidence of subtle host-associated differentiation (Table 4). When individuals were pooled by coral host taxon, global differentiation between samples was small but significant for the microsatellite data (*F*_ST_ = 0.002; *P*<0.001) and small and marginally significant for the mtDNA (Φ_ST_ = 0.007, *P* = 0.054; Table 4). Although the hierarchical AMOVA results differed slightly for the two types of molecular markers, depending on groupings, both also revealed small but significant effects of coral host and no significant effects of sampling locality on population structure (Table 4). After Bonferroni correction for multiple comparisons, however, only microsatellite-derived values remained significant.

**Table 4:** AMOVA results for tests of host-associated differentiation among populations of *Coralliophila abbreviata* using mtDNA and microsatellite (msats) data.

	Source of variation	Marker type	d.f.	S.S.	Fixation index	% var.	*P*-value
**i.**	Among host corals	mtDNA	1	1.05	Φ_ST_ = 0.0067	0.67	0.052
		msats	1	3.37	F_ST_ = 0.0017	0.17	**<0.001** *
**ii.**	Among populations (host x locality)	mtDNA	6	5.29	Φ_ST_ = 0.0103	1.03	**0.038**
		msats	8	19.35	F_ST_ = 0.0005	0.05	**<0.001** *
**iii.**	Among localities	mtDNA	3	2.50	Φ_CT_ = −0.0062	−0.62	0.753
		msats	3	7.53	F_CT_ = 0.0002	0.02	0.446
	Among host corals	mtDNA	3	2.80	Φ_SC_ = 0.0156	1.57	**0.016**
		msats	3	7.47	F_SC_ = 0.0011	0.12	0.369
**iv.**	Among host corals	mtDNA	1	1.02	Φ_CT_ = 0.0045	0.45	0.116
		msats	1	3.37	F_CT_ = 0.0018	0.18	**0.003** *
	Among localities within host corals	mtDNA	5	4.27	Φ_SC_ = 0.0077	0.77	0.134
		msats	7	15.98	F_SC_ = −0.0006	−0.06	0.864

Populations were defined by host taxon (i.: ACR, MON) or host and locality (ii.–iv.: FL ACR, FL MON, BAH ACR [msats only], NAV ACR, NAV MON, CUR ACR [msats only], CUR MON, SVG ACR, SVG MON). Results for microsatellite data represent the weighted averages over all loci.

Bold values were significant (α = 0.05) before Bonferroni correction for multiple comparisons,

* indicates significance after correction.

When the PAN MYC sample was included in the analysis and individuals were pooled by coral host taxon, no genetic structure was detected with the mtDNA (Φ_ST_ = 0.002, *P* = 0.23). However, subtle but significant genetic structure among hosts was still indicated based on microsatellite data (*F*_ST_ = 0.001, *P*<0.001). Subsequent pairwise comparisons indicated that the differentiation was driven by differences between ACR and MONT populations (MYC v. ACR: *F*_ST_ = 0.0014, *P* = 0.11; MYC v. MON: *F*_ST_ = −0.0016, *P* = 0.94; ACR v. MON: *F*_ST_ = 0.0014, *P* = 0.02).

Five pairwise comparisons derived from mtDNA (when populations were defined by host and locality) were significant (α = 0.05) before Bonferonni correction but none remained significant after correction and there were no significant pairwise comparisons derived from microsatellite data (Table 5). No significant effects of locality or oceanographic region (east versus west) were detected when host information was excluded (Table S2).

**Table 5:** Pairwise *F*_ST_ values for all populations of *Coralliophila abbreviata* defined by coral host and locality derived from microsatellite (below diagonal) and mtDNA (above diagonal) data.

	FL ACR	FL MON	BAH ACR	NAV ACR	NAV MON	SVG ACR	SVG MON	CUR ACR	CUR MON	PAN MYC
FL ACR		−0.006	NA	−0.015	0.023	0.020	−0.015	NA	−0.023	−0.025
FL MON	0.001		NA	0.011	0.016	0.004	0.000	NA	0.002	−0.005
BAH ACR	−0.025	−0.016		NA	NA	NA	NA	NA	NA	NA
NAV ACR	0.002	0.004	−0.013		**0.035**	**0.037**	0.015	NA	0.015	0.008
NAV MON	−0.002	−0.004	−0.009	0.006		**0.040**	0.007	NA	0.011	**0.026**
SVG ACR	−0.001	−0.001	−0.023	−0.002	−0.001		0.016	NA	**0.021**	0.017
SVG MON	−0.001	−0.001	−0.016	0.000	−0.004	0.001		NA	−0.017	−0.010
CUR ACR	−0.003	−0.002	−0.020	−0.001	−0.004	−0.005	−0.003		NA	NA
CUR MON	0.001	−0.002	−0.010	0.005	0.003	−0.002	−0.003	−0.004		−0.003
PAN MYC	−0.001	−0.002	−0.011	0.006	−0.003	−0.001	−0.001	−0.001	−0.004	

Bold values were significant (α = 0.05) before Bonferroni correction for multiple comparisons. No values remained significant after correction.

Overall, POWSIM simulations demonstrated that the microsatellite data contained sufficient power to detect low levels of population structure. Population structure was detected for simulated populations defined by 1.) host (two populations) when *F*_ST_ = 0.0013 with 92% accuracy, 2.) host and locality (ten populations) when *F*_ST_ = 0.0020 with 92% accuracy, 3.) locality (six populations) when *F*_ST_ = 0.0017 with 95% accuracy, and 4.) oceanographic region (two populations) when *F*_ST_ = 0.0012 with 91% accuracy. The finest resolution of structure tested in this study was between pairs of populations defined by host and locality, with an average sample size per population of 31. At this level, the microsatellite data set could detect an *F*_ST_ of 0.0074 with 91% accuracy in simulated populations.

Mitochondrial *cyt b* haplotypes did not cluster by coral host taxon or geographic region (Figure 2). There were no significant (α = 0.05) exact tests of population differentiation and we found no significant correlation between genetic and geographical distances (mtDNA: *r* = −0.299, *P* = 0.753; msats: *r* = −0.251, *P* = 0.828). Finally, for the STRUCTURE analysis, all individuals had approximately the same probability of originating from each cluster, regardless of the *K* value (Figure S1) and the lnP(K) was greatest for *K* = 1 with no overall trend across *K* values from 1–10, indicating that all individuals were sampled from a single population (Figure S2).

### Demographic History

The overall mismatch distribution was unimodal and significantly coincident with the distribution expected under the sudden demographic expansion model (Table 2; Figure S3). Based on the optimized value of τ (1.62), a generation time of 6 years, and mutation rates of 0.6% and 1.0% site^−1^ MY^−1^, the expansion began during the Pleistocene, approximately 219,000–365,000 years ago. Tajima's *D* and Fu's *F_S_* statistics were consistently negative and significantly different than expected under mutation-drift equilibrium (Table 2). The large negative values indicate an excess of rare alleles and a reduced number of common alleles, which is consistent with patterns expected as a result of a large population expansion or a selective sweep [47]–[49].

**Figure 3:**
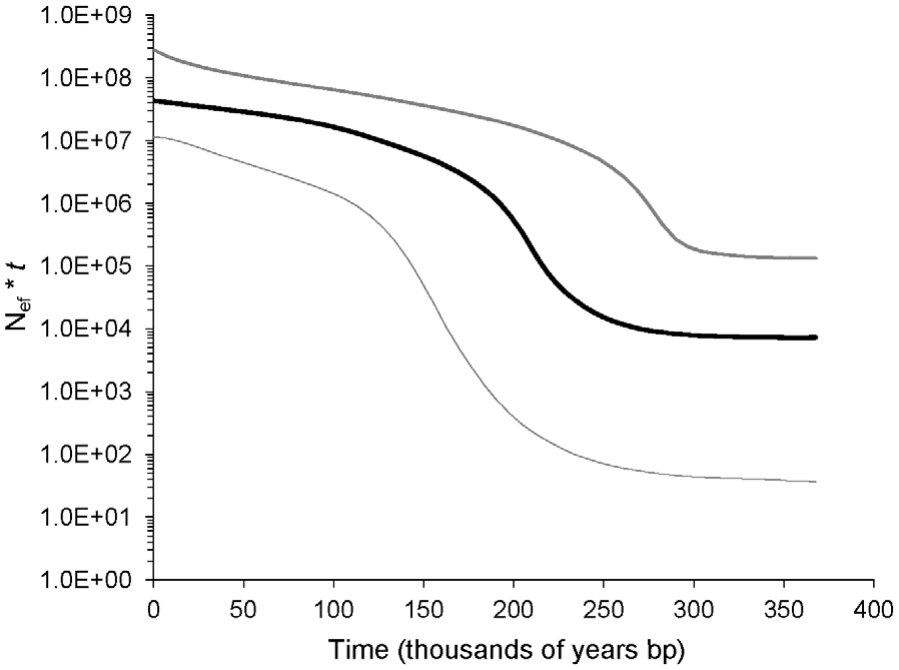
Bayesian skyline plot for *Coralliophila abbreviata* derived from mitochondrial cytochrome b sequence data. The solid black line represents the median female effective population size (*N*_ef_) multiplied by the generation time (*t*), plotted on a log scale. The thin grey lines are the upper and lower 95% highest posterior distribution (HPD) for the population size estimator.

The Bayesian skyline analysis implemented in BEAST indicated that the current median female effective population size is 7.2×10^6^ (Figure 3). The mean time since the most common recent ancestor (tMRCA) in the *cyt b* gene genealogy was 0.248 Ma (lower 95% HPD: 0.159 Ma; upper 95% HPD: 0.370 Ma), at which point a large population expansion began (Figure 3).

## Discussion

Genetic differentiation of sampled *C. abbreviata* populations was not detected at any geographical scale assessed in this study, including between populations separated by more than 3000 km. These results indicate that gene flow and connectivity are high across the species' range. High gene flow is consistent with expectations based on snail life-history characteristics, including high fecundity [24] and planktotrophic veliger larvae with a putative pelagic larval duration (PLD) of more than 30 days (Johnston, unpublished data). Although PLD has been decoupled from dispersal distance and gene flow in several Caribbean reef fishes [33], [54] and invertebrates [55], long distance dispersal and genetic homogeneity occur in other species of fish [54], [56]–[58] and invertebrates [59], [60] with moderate to high dispersal potential.

In addition to continued gene flow, large effective population sizes can maintain genetic homogeneity among populations over long periods of time after an expansion and subsequent demographic isolation. In such large populations, genetic drift has a smaller influence and it takes longer to reach drift-mutation equilibrium [61], [62]. Analysis of mtDNA suggests that *C. abbreviata* underwent a large population expansion during the Pleistocene (discussed below). Estimates of contemporary gene flow based on the mitochondrial sequence data should, therefore, be interpreted cautiously as genetic homogeneity may reflect historic rather than modern day demographic processes. Despite this caveat, it is probable that contemporary gene flow among *C. abbreviata* populations throughout the Caribbean is high enough to prevent significant genetic subdivision. We base this assertion on the observed lack of genetic structure across large spatial scales in both the mtDNA and microsatellite datasets (i.e. lack of IBD) and the life-history of the species (i.e. high dispersal potential via planktotrophic veliger larvae). Furthermore, coalescent-based estimates of ancestral population size indicate that demographic expansion occurred approximately 250,000 years ago (∼42,000 generations), providing ample time under most scenarios for allele frequencies to have diverged if gene flow had been restricted post-expansion [63].

Although high dispersal and gene flow appear to preclude local adaptation and diversification in many marine invertebrates [64], [65], there are several reports of host or habitat-associated differentiation at various spatial scales for marine organisms with moderate to high dispersal potential [7], [17],[33], [66]–[68]. Diversification in these cases appears to occur through disruptive selection acting on ecotypes and/or strong micro-habitat (e.g. host) settlement preference by larvae and fidelity by adults, resulting in assortative mating or reproductive isolation. Here, we found evidence of weak host-associated differentiation within *C. abbreviata* based on AMOVA analyses of microsatellite and mitochondrial *cyt b* sequence data (Table 4). However, patterns of differentiation may vary across geographical locations as the only pairwise comparison of host-associated snail populations within a sampling locality that approached significance was in Navassa, based on mitochondrial DNA sequence data (Table 5). Levels of differentiation for all other pairwise comparisons of host-associated populations within localities were small and non-significant, indicating a lack of host-associated genetic structure within these localities. However, in simulations based on current sampling effort and five microsatellite loci, genetic differentiation between two populations could only be detected with 90% or greater accuracy when *F*_ST_ values were greater than 0.007. Thus, levels of differentiation between host-associated populations within localities below *F*st = 0.007 may not have been detected.

The biological, ecological, and evolutionary importance of the observed low levels of host-associated differentiation is difficult to gauge based on current information [69]. It is possible that host-associated *C. abbreviata* populations are in the very early stages of divergence. Under recent divergence, even if contemporary gene flow is highly restricted or absent among host populations, the genetic signal may be weak or absent at neutral and nearly neutral loci due to mutation-drift processes. Alternatively, isolating mechanisms such as selection, host preference and fidelity might be too weak to overcome the homogenizing effects of gene flow and thus fail to form distinct host races or sibling species. Observational and experimental data tend to support this hypothesis. Although nothing is known about larval settlement behavior or very early life-history of *C. abbreviata*, snails will feed on multiple coral hosts in laboratory and field experiments [25] and there is some evidence that adult migration among host corals occurs on a reef scale. For instance, in the Florida Keys, adult snails have colonized transplanted *A. cervicornis* colonies in areas where other acroporid corals were not present (Miller, unpublished data). Also in the Florida Keys, individually tagged snails have been observed to move among and feed on multiple coral host taxa in a manipulative field experiment [70]. In some localities, however, it is plausible that isolating mechanisms are stronger due to other ecological and environmental factors, effectively reducing gene flow among hosts and resulting in the observed geographically variable patterns of host-associated differentiation.

### Adaptive Genetic Polymorphisms and Phenotypic Plasticity

Adaptive genetic polymorphisms may be maintained in a panmictic population through balancing selection when alternative genotypes exhibit greater fitness in specific habitats [71], [72]. Phenotypic plasticity, in which a single genotype can express multiple phenotypes under different environmental conditions, may also be adaptive across heterogeneous environments if the average net fitness across habitats is higher for the plastic genotype than for a specialist [73], [74]. However, phenotypic plasticity has associated fitness costs in terms of maintenance and imperfect phenotype to habitat matching among others [75]–[77] and may be lost over time due to assimilation of fitter specialist genotypes [78].

Baums et al. [25] conducted a reciprocal transplant experiment in which *C. abbreviata* snails were originally collected from both *A. palmata* and *Montastraea* spp. coral colonies. Regardless of the original coral host, recovered snails feeding on *A. plamata* grew faster than those feeding on *Montastraea* spp. corals. Thus, plasticity in snail growth under different environmental conditions (i.e. coral hosts) appears to contribute to the observed host-associated differences in population size structure. However, the relative importance of host-associated adaptation via genetic polymorphisms and phenotypic plasticity in the evolutionary ecology of *C. abbreviata* remains to be determined. These processes have important implications in regards to the propensity of *C. abbreviata* populations to diverge as coral community structure and other environmental conditions change across space and time. Further research is thus needed to fully assess the potential influence of selection acting on genotypes when occupying diverse coral hosts.

### Demographic History

Since very little genetic substructure was observed, we combined all samples to assess the population demographic history of *C. abbreviata* in the Caribbean. The mtDNA-based analyses consistently supported a scenario of Pleistocene demographic expansion preceded by a reduction in population size for *C. abbreviata*. The shallow *cyt b* gene genealogy with a single dominant haplotype and many new mutations (singleton haplotypes), resulting in moderate/high haplotype diversity (*h* = 0.773) and low nucleotide diversity (*π* = 0.4%) suggested a single colonization/founder event or a selective sweep followed by a rapid demographic expansion. Based on the mismatch distribution, the expansion began during the Pleistocene, approximately 219,000–365,000 years ago. This time frame was in agreement with the mean tMRCA and onset of expansion (∼250,000 years ago) determined through Bayesian skyline analysis (Figure 3).

The Plio-Pleistocene was a time of faunal turnover and subsequent changes in the diversity and structure of Caribbean corals reefs. After a late Pliocene/early Pleistocene extinction of scleractinian corals (4–1.5 Ma; [79]), there was an ecological shift from small, free-living species to a few large reef building species [80], [81]. The *Acropora* spp. and *Montastraea* spp. corals in particular achieved ecological dominance during the Pleistocene and remained dominant through recent geological time [80]–[83].

Sea level fluctuations during Pleistocene glacial cycles isolated basins and altered current patterns leading to demographic contractions and expansions in tropical marine taxa across the Indo-Pacific [84]–[87] and to a lesser extent in the Caribbean and tropical western Atlantic [54]. Our data, however, indicate that *C. abbreviata* populations persisted in high numbers through the last glacial maxima (∼20,000 years ago). We hypothesize that *C. abbreviata* colonized the greater Caribbean region during the mid-late Pleistocene and subsequently expanded with the expansion of reef habitat and potential prey. Indeed, Johnson et al [88] reported that the widespread increase in carbonate reef development following the phase shift in coral community structure led to an increase in the diversity of reef associated mollusks during the Pleistocene to recent. Additionally, Plio-Pleistocene invasions from the Indo-Pacific and eastern Atlantic to the Caribbean and western Atlantic have been demonstrated for several species of fish [54], [89],[90] and at least 33 species of mollusks [91]. The colonization success and diversification of mollusks during this time is thought to be due to the large scale expansion of reef habitat [88], [91].

### Conclusions

We used five highly polymorphic microsatellite loci and 366 bp of the mitochondrial cytochrome b gene to assess the population genetic structure, connectivity, and demographic history of *Coralliophila abbreviata* sampled from different host coral species and geographic localities. Although *C. abbreviata* have not diverged into host races or sibling species, we found evidence of weak host-associated differentiation. *Coralliophila abbreviata* populations exhibited stronger differentiation by coral host taxon than by geographic locality. The biological, ecological, and evolutionary importance of the observed extremely low levels of host-associated differentiation remains to be seen. Overall, the results of this study indicate that *C. abbreviata* constitutes a large, interconnected meta-population throughout the greater Caribbean that expanded during the Pleistocene, likely due to the large-scale expansion of reef habitat during that time. However, direct field-based demographic surveys and experiments as well as additional molecular studies are needed to determine the magnitude and ecological importance of dispersal and connectivity among coral hosts. Future molecular studies should be based on extensive sampling of sympatric host-associated populations across multiple spatial scales using additional neutral and adaptive markers to fully elucidate the interactive effects of host and locality on the population genetic structure, ecology, and evolution of *C. abbreviata*.

### Implications for Coral Reef Conservation

The *Acropora* spp. corals have declined drastically throughout the Caribbean over the last three decades due to a variety of natural and anthropogenic stressors, resulting in their listing as ‘Threatened’ species under the U.S. Endangered Species Act in 2006. Predation by *C. abbreviata* may represent a profound threat to the persistence and recovery of remnant populations of the Caribbean acroporids [92]–[95]. Understanding the patterns of host-use and connectivity of *C. abbreviata* is thus important for coral conservation efforts. Whereas Baums et al. [32] identified regionally isolated populations of the host coral *A. palmata*, the results of this study indicate that gene flow is high across the Caribbean for *C. abbreviata*. Thus, snail populations are decoupled demographically from local and regional population fluctuations of the threatened acroporid corals as snails may be supplied from distant locations and maintained on alternative coral prey. Targeted snail removal, therefore, may be necessary to ensure the persistence and/or recovery of particularly vulnerable *Acropora* colonies such as small fragments or remnant colonies, nursery transplanted colonies and new recruits. However, removal efforts may be offset by input from other local and regional sources and any potential control strategies need to be designed accordingly.

## Supporting Information

Supporting Information S1 **Detailed methodology for microsatellite development, amplification, and testing.**
(PDF)

Table S1 **Sample sizes of** ***Coralliophila abbreviata*** **by region, locality and reef for mitochondrial** ***cyt b*** **sequences (mtDNA) and microsatellite markers (Msats) with sampling coordinates.**
(PDF)

Table S2 **AMOVA results for tests of geographic differentiation among sampled populations of** ***Coralliophila abbreviata*** **.**
(PDF)

Figure S1 **STRUCTURE plots representing the probability of membership to each hypothetic population for 311 individual** ***Coralliophila abbreviata*** **based on five polymorphic microsatellite loci.** Shown are plots for K = 2 (a), K = 4 (b), and K = 10 (c), where individuals are grouped by sampling locality (1: Bahamas; 2: Curacao; 3: Florida; 4: Navassa; 5: Panama; 6: St. Vincent and the Grenadines).
(PDF)

Figure S2 **STRUCTURE results: mean (±SD) of estimated Ln probability of the data for each K value.**
(PDF)

Figure S3 **Mismatch distribution.** The observed number of pairwise nucleotide differences (open circles) for mitochondrial *cyt b* sequences plotted with the expected number of pairwise nucleotide differences under a model of sudden demographic expansion (solid line) and the 95% confidence intervals for the model estimation (dashed lines).
(PDF)
