# Supplementary material for: Assessment of Host-Associated Genetic Differentiation among Phenotypically Divergent Populations of a Coral-Eating Gastropod across the Caribbean
Source: PLoS One. 2012 Nov 2;7(11):e47630. doi: 10.1371/journal.pone.0047630 (PMC3487833; doi:10.1371/journal.pone.0047630)
Supplement: Supporting Information S1 — Detailed methodology for microsatellite development, amplification, and testing. (PDF) [file pone.0047630.s001.pdf]

Supporting Information S1. Detailed methodology for microsatellite development, amplification, and testing.

Genomic DNA from two individual *C. abbreviata* was enriched for di-, tri-, and tetra-nucleotide repeat motifs and used to create a cDNA library using methods modified from Glenn and Schable Glenn and Schable (2005). DNA samples were digested using the restriction enzymes *Rsa*I and *Bst*UI. DNA fragments were then ligated to a double stranded linker (SuperSNX24 Forward: 5'-GTTTAAGGCCTAGCTAGCAGAATC-3' and SuperSNX24+4P Reverse: 5'-pGATTCTGCTAGCTAGGCCTTAAACAAAA-3') with DNA ligase at16 °C. The linker-ligated DNA fragments were then hybridized to the 3' biotinylated microsatellite probes. Enriched DNA fragments were captured using Dynabeads (Invitrogen), amplified by PCR using the SuperSNX24 forward primer (5'-GTTTAAGGCCTAGCTAGCAGAATC-3'), and cloned using a TOPO TA Cloning System (2.1; Invitrogen), following the manufacturer's instructions. Positive colonies were amplified by PCR using universal M13 forward and reverse primers. PCR products in the 500-1000 bp size-range were then purified and sequenced using an ABI 3730 automated sequencer. Sequences were assembled and edited in SEQUENCHER v4.1 (Gene Codes Corp) and then visually searched for microsatellite repeats. Thirteen primer pairs were designed for microsatellite flanking regions using Primer3 software (Rozen and Skaletsky1998; Code available at http://www-genome.wi.mit.edu/genome_software/other/primer3.html). In initial test using 24 individuals, 11 of the 13 loci amplified products of the expected size and eight were polymorphic. The eight polymorphic loci were then labeled with one of four fluorescent dyes (6-FAM, HEX, NED, VIC; Applied Biosystems) on the forward primer. PCR amplifications were optimized and loci were further characterized in a sample of 60 individual *C. abbreviata* collected from the Florida Keys National Marine Sanctuary in the western Atlantic (n = 31) and St. Vincent and the Grenadines in the Eastern Caribbean (n = 29).

For each microsatellite locus, the number of observed alleles, allele frequencies, and observed and expected heterozygosity were determined using the program GENEPOP v.4 RAYMOND and ROUSSET (1995). GENEPOP was also used to calculate F_{IS} values and test for deviations from Hardy-Weinberg equilibrium (HWE) and linkage equilibrium for each locus. All microsatellite loci were checked for the presence of null alleles and errors due to stuttering and large allele dropout using the program MICRO-CHECKER VAN OOSTERHOUT et al. (2004)

No linkage disequilibrium between pairs of loci was detected ($P > 0.05$). After initial characterization of the eight loci in a subset of 60 individuals from two localities, three loci (Ca602, Ca606, and Ca607) deviated significantly from HWE due to large heterozygote deficits in both populations after Bonferonni correction for multiple comparisons ($P < 0.001$). Heterozygote deficiencies in microsatellite loci can result from several processes, including technical amplification and scoring errors such as large allele drop out and stuttering, as well as the presence of null alleles, population structure, and inbreeding. The effects of population structure and inbreeding generally manifest across all loci and can, therefore, tentatively be ruled out here. Errors due to stuttering and large allele dropout were not detected for any locus using MICROCHECKER. Significant frequencies of null alleles, however, were detected for all three of the loci that deviated from HWE ($P < 0.05$). High frequencies of null alleles can introduce errors in some genetic estimates, thus, these three loci were not used in further analyses.

Optimized PCR amplifications were carried out in a 10µL reaction volume containing 50-100 ng of genomic DNA, 1X PCR buffer (containing 1.5 mM $MgCl_2$; New England Biolabs), 0.2 mM of each dNTP, 0.15 µM 5'-labeled forward primer, 0.15 µM unlabelled reverse primer, and 1 U Taq DNA polymerase (New England Biolabs). The following touchdown thermal

cycling program was used: 94 °C for five min, followed by three cycles of 94 °C for 15 s, 60 °C for 15 s, 72 °C for 45 s, 12 cycles of 94 °C for 15 s, 60 °C - 54 °C (ramping down 0.5 °C per cycle) for 15 s, 72 °C for 45 s, 25 cycles of 94 °C for 15 s, 54 °C for 15 s, 72 °C for 45 s, and finally 72 °C for 10 min. The PCR products were separated on an ABI 3730 sequencer with an internal size standard to ensure accurate sizing (Gene Scan 500LIZ, Applied Biosystems) and alleles were then scored from electropherograms using GENEMAPPER v.4 software (AppliedBiosystems).

References

Glenn TC, Schable NA (2005) Isolating microsatellite DNA loci Meth Enzymol 395:202-222

Raymond M, Rousset F (1995) GENEPOP (version 1.2) Population genetics software for exact tests and ecumenicism. J Hered 86:248-249

van Oosterhout C, Hutchinson WF, Wills DPM, Shipley P (2004) Micro-checker: Software for identifying and correcting genotyping errors in microsatellite data. Molecular Ecology Notes 4:535-538 DOI 10.1111/j.1471-8286.2004.00684.x
