# Supplementary material for: Assessment of Host-Associated Genetic Differentiation among Phenotypically Divergent Populations of a Coral-Eating Gastropod across the Caribbean
Source: PLoS One. 2012 Nov 2;7(11):e47630. doi: 10.1371/journal.pone.0047630 (PMC3487833; doi:10.1371/journal.pone.0047630)
Supplement: Table S1 — Sample sizes of Coralliophila abbreviata by region, locality and reef for mitochondrial cyt b sequences (mtDNA) and microsatellite markers (Msats) with sampling coordinates. (PDF) [file pone.0047630.s002.pdf]

Table S1. Sample sizes of *Coralliophila abbreviata* by region, locality and reef and sampling coordinates for mitochondrial *cyt b* sequences (mtDNA) and microsatellite markers (Msats).

Region	Locality	Reef name	Lat (°)	Long (°)	mtDNA	Msats
**West**	Florida	Little Grecian	25.1184	-80.3171	27 (A:12; M:15)	56 (A:24;M32)
		Sand Island	25.0179	-80.3686	-	15 (A:13;M:2)
		Florida Total			27 (A:12; M:15)	71 (A:37;M:34)
	Bahamas	Green Turtle Cay	26.7667	-77.3167	-	12 (A:12)
	Navassa	NW Point	18.4138	-75.0297	6 (M:6)	9 (M:9)
		W Pinnacles	18.4047	-75.0267	10 (A:4; M:6)	14 (A:6; M:8)
		DOT 118	18.3962	-75.0189	12 (A:12)	10 (A:10)
		Navassa Total			28 (A:16; M:12)	33 (A:16; M:17)
	Panama	Hospital Pt.	9.3380	-82.2407	30 (MY:30)	51 (MY:51)
	West Total				85 (A:28; M:27;MY:30)	167 (A:65; M:51;MY:51)
**East**	SVG	Blue Lagoon	13.1285	-61.1993	5 (A:5)	15 (A:15)
		Bequia	13.0150	-61.2491	16 (A:2; M:14)	33 (A:7; M:26)
		Conouan	12.6943	-61.3364	3 (A:3)	11 (A:5; M:6)
		Mustique	12.8915	-61.1863	3 (A:3)	8 (A:8)
		Tobago Cay	12.6253	-61.3499	-	12 (M:12)
		Union Island	12.5916	-61.4160	-	1 (A:1)
		SVG Total			27 (A:13; M:14)	80 (A:36; M:44)
	Bonaire	Taylors Made	12.2238	-68.4051	-	16 (A:7; M:9)
	Curacao	Awa Blanca	12.0406	-68.7834	-	2 (A:2)
		Blue Bay	12.1352	-68.9898	4 (M:4)	10 (A:2; M:8)
		Playa Largu	12.1470	-69.5540	10 (M:10)	21 (A:11; M:10)
		Sea Aquarium	12.0838	-68.8958	4 (M:4)	13 (A:3; M:10)
		Curacao/Bonaire Total			18 (M:18)	46 (A:25; M:39)
	East Total				45 (A:13; M:32)	142 (A:61; M:81)
**TOTAL**					130	309
