# Supplementary material for: Assessment of Host-Associated Genetic Differentiation among Phenotypically Divergent Populations of a Coral-Eating Gastropod across the Caribbean
Source: PLoS One. 2012 Nov 2;7(11):e47630. doi: 10.1371/journal.pone.0047630 (PMC3487833; doi:10.1371/journal.pone.0047630)
Supplement: Table S2 — AMOVA results for tests of geographic differentiation among sampled populations of Coralliophila abbreviata . (PDF) [file pone.0047630.s003.pdf]

Table S2. AMOVA results for tests of geographic differentiation among sampled populations of *Coralliophila abbreviata*.

	Source of variation	Marker type	d.f.	S.S.	Fixation index	% var.	*P*-value
**i.**	Between regions (east, west)	mtDNA	1	0.91	Φ_{ST}=0.0027	0.27	0.192
		msats	1	2.27	F_{ST}=-0.0001	-0.01	0.636
**ii.**	Among localities	mtDNA	4	3.24	Φ_{ST}=0.0012	0.12	0.354
		msats	5	11.55	F_{ST}=-0.0002	-0.02	0.754
**iii.**	Among regions	mtDNA	1	0.91	Φ_{CT}= 0.0029	0.29	0.301
		msats	1	2.27	F_{CT}= -0.0000	-0.00	0.371
	Among localities within regions	mtDNA	3	2.32	Φ_{SC}= -0.0005	-0.05	0.518
		msats	4	9.28	F_{SC}= -0.0002	-0.02	0.712
