# Supplementary material for: Assessment of Host-Associated Genetic Differentiation among Phenotypically Divergent Populations of a Coral-Eating Gastropod across the Caribbean
Source: PLoS One. 2012 Nov 2;7(11):e47630. doi: 10.1371/journal.pone.0047630 (PMC3487833; doi:10.1371/journal.pone.0047630)
Supplement: Figure S1 — STRUCTURE plots representing the probability of membership to each hypothetic population for 311 individual Coralliophila abbreviata based on five polymorphic microsatellite loci. Shown are plots for K = 2 (a), K = 4 (b), and K = 10 (c), where individuals are grouped by sampling locality (1: Bahamas; 2: Curacao; 3: Florida; 4: Navassa; 5: Panama; 6: St. Vincent and the Grenadines). (PDF) [file pone.0047630.s004.pdf]

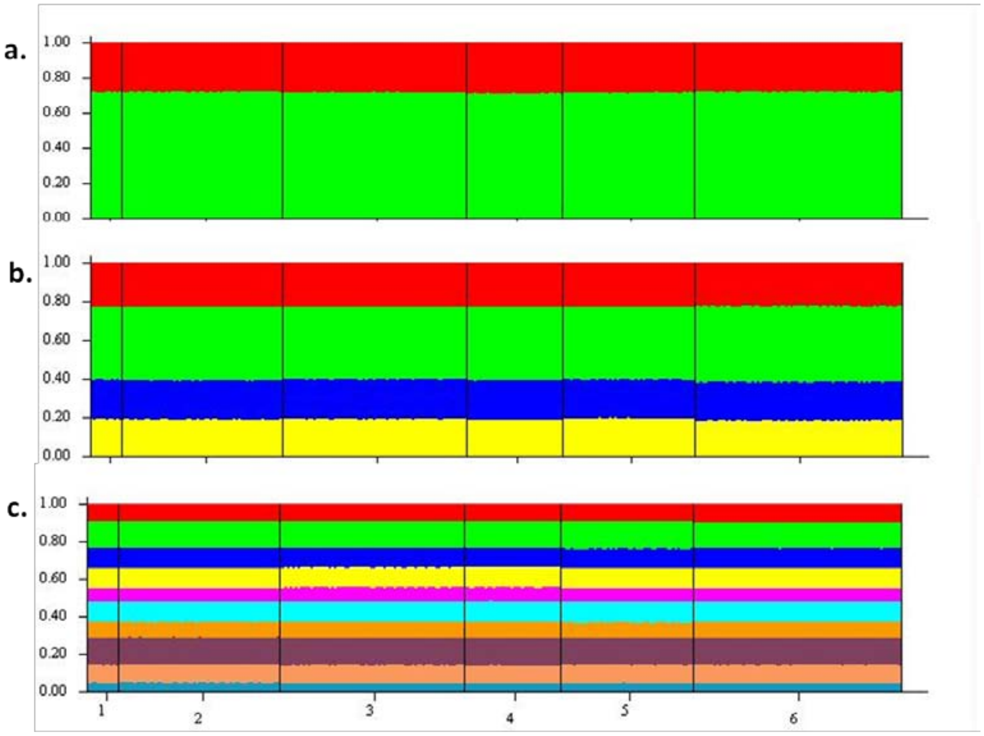

Figure S1. STRUCTURE plots representing the probability of membership to each hypothetic population for 311 individual *Coralliophila abbreviata* based on five polymorphic microsatellite loci. Shown are plots for K = 2 (a), K = 4 (b), and K = 10 (c), where individuals are grouped by sampling locality (1: Bahamas; 2: Curacao; 3: Florida; 4: Navassa; 5: Panama; 6: St. Vincent and the Grenadines).
