# Supplementary figures and images for: Assessment of Host-Associated Genetic Differentiation among Phenotypically Divergent Populations of a Coral-Eating Gastropod across the Caribbean
Source: PLoS One. 2012 Nov 2;7(11):e47630. doi: 10.1371/journal.pone.0047630 (PMC3487833; doi:10.1371/journal.pone.0047630)

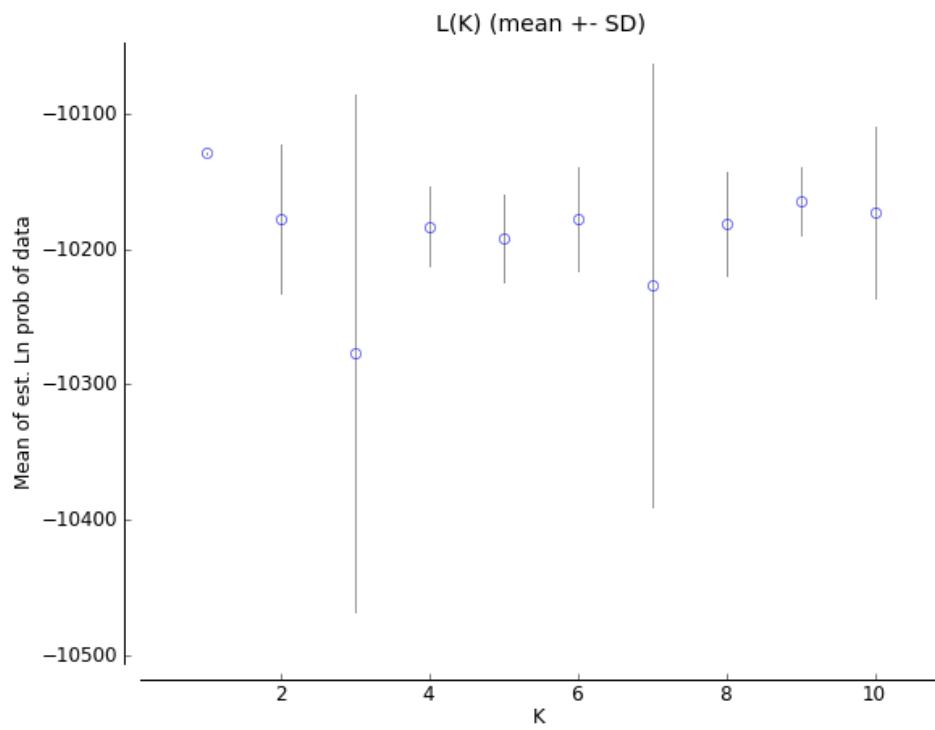

**Figure S2.** STRUCTURE results: mean ( $\pm$ SD) of estimated Ln probability of the data for each K value.

Supplement: Figure S2 — STRUCTURE results: mean (±SD) of estimated Ln probability of the data for each K value. (PDF) [file pone.0047630.s005.pdf]
