# Supplementary material for: Assessment of Host-Associated Genetic Differentiation among Phenotypically Divergent Populations of a Coral-Eating Gastropod across the Caribbean
Source: PLoS One. 2012 Nov 2;7(11):e47630. doi: 10.1371/journal.pone.0047630 (PMC3487833; doi:10.1371/journal.pone.0047630)
Supplement: Figure S3 — Mismatch distribution. The observed number of pairwise nucleotide differences (open circles) for mitochondrial cyt b sequences plotted with the expected number of pairwise nucleotide differences under a model of sudden demographic expansion (solid line) and the 95% confidence intervals for the model estimation (dashed lines). (PDF) [file pone.0047630.s006.pdf]

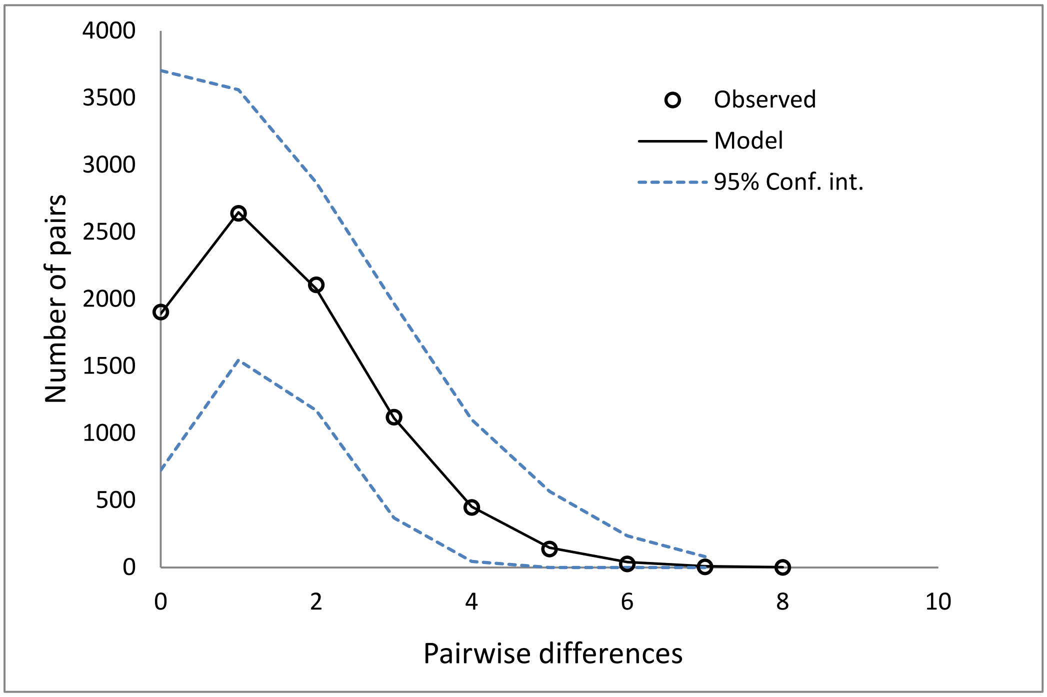


**Figure S3.** Mismatch distribution. The observed number of pairwise nucleotide differences (open circles) for mitochondrial *cyt b* sequences plotted with the expected number of pairwise nucleotide differences under a model of sudden demographic expansion (solid line) and the 95% confidence intervals for the model estimation (dashed lines).
